# Supplementary material for: Farnesol Emulsion as an Effective Broad-Spectrum Agent against ESKAPE Biofilms
Source: Antibiotics (Basel). 2024 Aug 17;13(8):778. doi: 10.3390/antibiotics13080778 (PMC11352207; doi:10.3390/antibiotics13080778)
Supplement: Supplementary file 1 [file antibiotics-13-00778-s001.zip › antibiotics-3118690-supplementary.pdf]

Supplementary Materials:

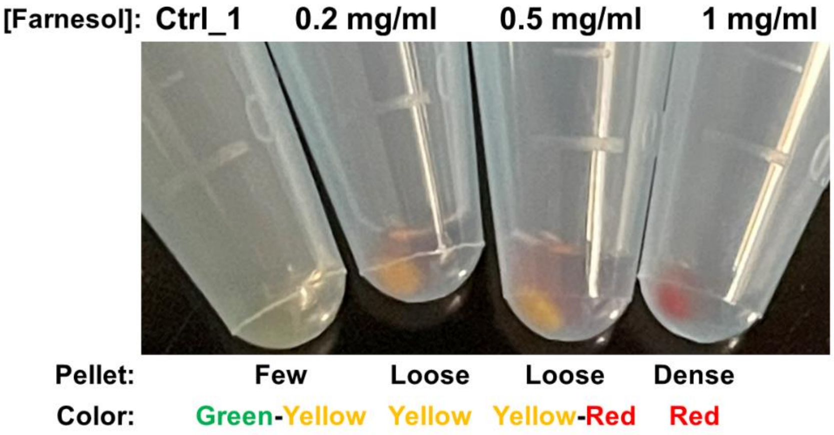

Figure S1. Farnesol detaches *E. faecium* established biofilm to generate detached cells. The supernatants containing detached *E. faecium* cells after farnesol treatment were centrifuged, and the obtained pellets were progressively dense (and easier to collect by centrifuging) with increased farnesol doses**.** After assayed with the Live/Dead viability kit, the pellets also showed gradual color changes from green to yellow, then to red, with increased farnesol doses. Ctrl_1 = 3.3% of ethanol.

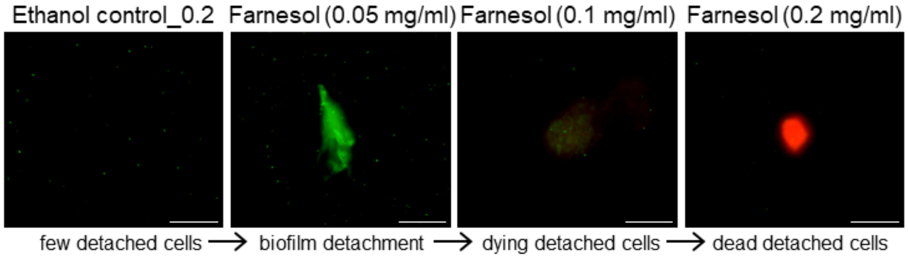

Figure S2. Farnesol combats *E. cloacae* by direct killing and biofilm detachment. The supernatant containing detached *E. cloacae* cells after 24-h exposure of farnesol treatment were centrifuged, and the obtained pellet was

then washed and stained with both SYTO® 9 (green fluorescence for live cells) and PI (red fluorescence for dead cells). The obtained merged signals were displayed side-by-side. Scale bars, 20 μm. Control_0.2 = 0.67% of ethanol.

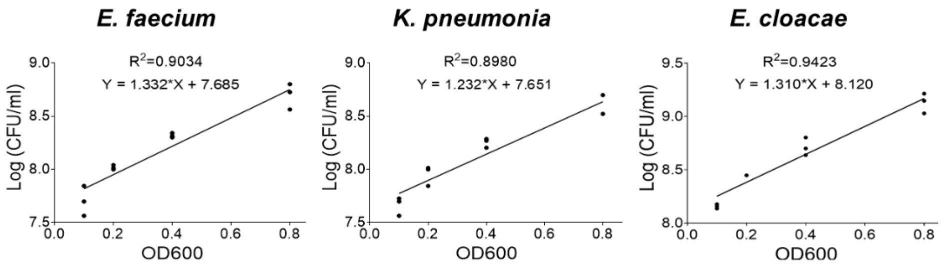

Figure S3. Formula for conversion between OD600 and CFU/ml for *E. faecium* (BAA-2316), *K. pneumoniae* (BAA-2146) and *E. cloacae* (BAA-2468) strains used in the study. The effective linear range of OD600 is 0.1 to 0.8. The coefficient of determination (R^2) values for each formula are shown at the top of each panels.
